# Efficient multiuser quantum cryptography network based on entanglement

**DOI:** 10.1038/srep45928

**Published:** 2017-04-04

**Authors:** Peng Xue, Kunkun Wang, Xiaoping Wang

**Affiliations:** 1 Department of Physics, Southeast University, Nanjing 211189, China; 2 State Key Laboratory of Precision Spectroscopy, East China Normal University, Shanghai 200062, China

## Abstract

We present an efficient quantum key distribution protocol with a certain entangled state to solve a special cryptographic task. Also, we provide a proof of security of this protocol by generalizing the proof of modified of Lo-Chau scheme. Based on this two-user scheme, a quantum cryptography network protocol is proposed without any quantum memory.

Suppose Alice, a mother, wishes to control the correspondence among her son Bob and his friends. It means that if and only if attained her permissibility, Bob can correspond with his friends. However, the fellows’ communication is wanted to be secret to Alice. What can they do?

Classical cryptography provides an answer that is known as cryptography network. The primary task of cryptography is to enable two or more parties to mask confidential messages, such that the transmitted data are illegible to any unauthorized party, an eavesdropper called Eve. Usually this is done by using shared secret keys. However, in classical physics, there is nothing to prevent an eavesdropper from monitoring the key distribution channel passively, without being caught by the legitimate users. The recent development of quantum key distribution (QKD)^1^,^2^,^3^,^4^,^5^,^6^,^7^,^8^,^9^,^10^,^11^,^12^ can cover this major loophole of classical cryptography. It is based on the fundamental postulate of quantum physics that “every measurement perturbs a system”.

The first scheme for quantum cryptography, presented by Bennett and Brassard^1^, uses four states of single photons polarized along different directions, and various other types of quantum crypto-system have since been suggested. The other well-known concept for QKD is the Ekert scheme^2^ which is based on entangled pairs and uses the generalized Bell’s inequality^13^,^14^ to establish security. There are variant schemes utilizing Einstein-Podolsky-Rosen (EPR) correlations^6^,^15^, and proposed schemes based on two nonorthogonal states^4^, a variate of the four-state scheme^16^,^17^ and some ones based on orthogonal states^18^,^19^. Furthermore, some have been demonstrated experimentally^20^,^21^,^22^,^23^,^24^,^25^,^26^,^27^,^28^,^29^,^30^,^31^.

There are also theoretical proposals for QKD between more than two parties based on Greenberger-Horne-Zeilinger (GHZ) states^32^,^33^, and a first experiment has already been performed^34^.

If some practical techniques were to become widespread, however, it would have to be effective over a quantum cryptography network. Bihan *et al*.^35^ proposed the time-reserved EPR protocol which combined with quantum memories to build a quantum cryptography network. In a series of publications Townsend *et al*.^36^ have shown how the properties of passive optical networks (PONs) can be exploited to give one-to-any and any-to-any key distribution using quantum cryptography on branch- and loop-configuration networks. Recently, we have presented a conditional efficient multiuser quantum cryptography network scheme^37^ with three nonorthogonal states, which involves EPR source, a quantum random number generator and optical switches, and therefore well fits the status of the current experimental technology.

In this paper, we propose a QKD scheme with a certain entanglement of three qubits to solve the above-mentioned special cryptographic task. Combined with the idea presented by Lo *et al*.^17^, the efficiency of this scheme can be increased to tend to 100%. Suppose the center Alice creates pairs of particles in the entangled state |*ABC*〉 (seeing below), and sends a sequence of particles B and C out of each pairs to the two users Bob and Carol, and the corresponding particles A are left to herself. They choose their bases independently with different probabilities and perform measurements on their particles. And then, Alice broadcasts the results of measurements, which the two users announce their bases actually chosen via the classical channel to establish a common key between them. As two parties are much more likely to be using the same basis, thus reducing the fraction of discarded data, a significant gain in efficiency is achieved. To ensure our scheme is secure, we divide the accepted data into various subsets according to the bases employed and estimate an error rate for each subset separately. We then show that such a refined error analysis is sufficient in ensuring the security of our scheme against a biased eavesdropping strategy. The proof is based on the technique in Shor-Preskill’s proof of the security of another schemes^38^. Remarkably, we can establish a multi-user quantum cryptography network based on this scheme without quantum memory.

This paper is organized as follows. In the next section, an efficient two-user QKD scheme is proposed with a certain entanglement of three qubits. By considering a simple biased eavesdropping strategy by Eve, we note that analysis is sufficient in ensuring the security against this eavesdropping attack. Then, we consider the most general type of eavesdropping strategy allowed by quantum mechanics and prove that our scheme is unconditional secure. In addition, the constraint on the probability is derived. In Sec. III, using a series of certain entanglement, a quantum cryptography network is established based on the QKD scheme. Finally, we give a brief discussion and conclusion in Sec. IV.

## Results

### Efficient Two-User QKD Scheme

We choose a two-user scenario by way of example (See Table 1). In our scheme, there are three parties: the center, Alice and the users, Bob and Carol. Alice prepares pairs of particles in a certain entangled state


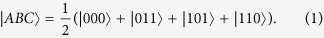


Particles B and C are sent to Bob and Carol, and the corresponding particle A is left for herself. There are two types of measurements that the receivers may perform: they may measure along the rectilinear basis, thus distinguishing between particles in the states |0〉 and |1〉. Alternatively, they may measure along the diagonal basis, thus distinguishing between the states 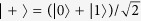 and 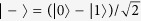.

Three parties are connected by a quantum channel and a classical public channel. The quantum channel consists usually of an optical fiber. The public channel, however, can be any communication link. So how does this scheme work?

1. Alice, Bob, and Carol pick a number 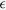, 0 < 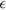 ≤ 1 and make it value public. The constraint on 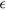 will be discussed in Part D.
2. Alice prepares a sequence of pairs of particles in the entangled state |*ABC*〉, and sends particles B to Bob and particles C to Carol, and the corresponding particles A for herself.
3. If she allows the communications between the two users, Alice performs measurements along rectilinear basis (i.e., {|0〉, |1〉}), which can yield two possible results 0 and 1, therefore can potentially reveal one bit of information, and then registers the outcome of the measurements.
4. For each particle, Bob (Carol) has two types of measurements. One measurement is along rectilinear basis, and the other is along diagonal basis (i.e., 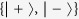). He chooses between the two types with probabilities 1 − 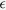 and 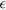, respectively. If he detects particle B (C) in the state |0〉 or 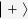, the result is 0; else, the measurement yields the result 1, and potentially reveals one bit of information. He writes down his measurement bases and the results of the measurements. The ensemble of these bits registered by both Bob and Carol is the raw key.
5. After exchanging enough particles, Alice broadcasts over the public channel the results based on her measurements.
6. Now Bob and Carol tell each other the sequence of bases they used, but not the results that they obtained, and keep only the bits corresponding to the same basis. There are two cases in which Alice obtains the measurement result 0 or 1. For each of these cases, both Bob and Carol are much more likely to choose the rectilinear basis and obtain correlated bits, thus achieving a significant gain in efficiency. This is a modified Ekert QKD scheme between Bob and Carol. If Alice yields “1”, the two users share a pair of particles in one of the EPR state 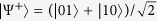. Because of the anti-correlations, in order to obtain identical keys, either of them should invert all bits of the key. Else, the pair of particles is in the state 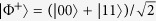. Therefore, they would not do anything. Obviously, the key is secret to the center. In one word, the sifted key is generated with the total probability (1 − 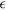)^2^ + 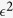 which goes to 1 as 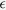 goes to zero. However, due to imperfections in the transmission, and to a potential eavesdropper, there will be some errors.
7. Bob and Carol throw away the useless cases when they have used incompatible bases. Since the total probabilities for the two users to obtain the results “0” and “1” are equal, the ensemble of these bits of the remaining cases is a sifted key. Therefore, the remaining cases are kept for further analysis and to generate the secret key.
8. Bob and Carol divide up the accepted data into two subsets according to the actual bases. In one subset where the two users both use the rectilinear basis, they randomly pick a fixed number say *m*_1_ particles and publicly compare their measurement results. The number of mismatches *r*_1_ (here, mismatch means the bit values of measurements are not correlated) tells them the estimated error rate 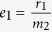. Similarly, in the other subset where they both use the diagonal basis, they pick a fixed number say m_2_ particles and publicly compare their measurement results. The number of mismatches *r*_2_ gives the estimated error rate 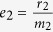. Note that the test samples *m*_1_ and *m*_2_ are sufficiently large, the estimated error rates *e*_1_ and *e*_2_ should be rather accurate. Now they demand that *e*_1_ and *e*_2_ < *e*_*max*_ where *e*_*max*_ is a prescribed maximal tolerable error rate. If these independent constraints are satisfied, they proceed to the next steps. Otherwise, they throw away the bit values of measurement and re-start the whole procedure. Notice that the constraints *e*_1_ and *e*_2_ < *e*_*max*_ are more stringent than the original naive prescription 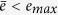 (here 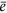 is the average error rate). We will discuss it in detail in Part B.
9. If the error rates are not too high, they can use classical information processing techniques, such as error correlation and privacy amplification (seeing ref. 38), to reduce the error rates to zero, while reducing the information obtained by Eve to zero as well.

From the discussion in the step 6, we know that the efficiency of the sifted key can tend to 100%. However, after some classical error correction and privacy amplification, the efficiency of the secret key cannot achieve 100%, and it depends on the error rate which is generated by both eavesdropping and intrinsic noise due to experimental imperfections. Suppose we use a classical linear code *C(k, N, d*) with *N* bits, having 2^*k*^ code words and minimum distance *d* as an error correction^39^. And the code of minimum distance *d* > 2*t* is necessary if *t* errors are to be corrected. In what follows, we will make use of two simple bounds, the Hamming or sphere-packing bound introduced by Hamming in 1950 and the Gilbert-Varshamov bound. In the limit of large *N*, it takes the form


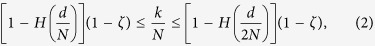


where *ζ* → 0 as *N* → ∞, and *H(x*) is the entropy function


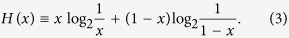


So the secret key’s rate approaches 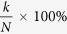, and still more efficient than that of other schemes (For example, shown in ref. 33, the efficiency after error correction tends to 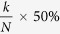).

For each particle, as the choices of bases used by Bob and Carol are unknown to the eavesdropper, Eve, any interaction by her will unavoidably modify the transmission and introduce some errors. She has eavesdropping attack as below: (i) with a probability *p*_1_(*p*_2_) measures the state along the rectilinear (diagonal) basis and re-sends a particle according to the result of her measurement to the user; (ii) with a probability 1 − *p*_1_ − *p*_2_ does nothing.

Consider the error rate *e*_1_ for the case where both Bob and Carol use the rectilinear basis. For the biased eaves-dropping strategy under current consideration, errors occur only if Eve uses the diagonal basis. This happens with a *conditional* probability *p*_2_. In this case, the polarization of the particle is randomized, thus giving an error rate is


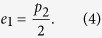


Similarly, errors for the case where both Bob and Carol use the diagonal basis happen with a conditional probability *p*_1_. Thus, the error rate for this case is given


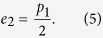


Therefore, the users will find that, for the biased eavesdropping attack, the average error rate


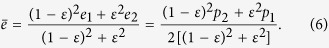


Suppose Eve always eavesdrops only along rectilinear basis (i.e., *p*_1_ = 1, *p*_2_ = 0), then


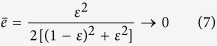


as 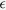 tends to 0, which is similar with the result of ref. 17. This means that if Eve is always eavesdropping along the rectilinear basis, with a naive error analysis prescribed as *e* < *e*_*max*_ Bob and Carol will fail to detect eavesdropping by Eve.

To ensure the security of our scheme, it is crucial to employ a refined data analysis: the accepted data are further divided into various subsets according to the actual bases, and the error rate of each subset is computed separately. From Eqs (4 and 5), we can see that these error rates *e*_1_ and *e*_2_ depend on Eve’s eavesdropping strategy, but not on the value of 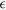. So the refined data analysis guarantees the security of the present scheme against the biased eavesdropping attack.

In this part, we provide a proof of security of our QKD scheme against the most general type of attack that is allowed by quantum mechanics, by generalizing the proof of modified Lo-Chau EPR scheme proposed by Shor and Preskill^38^, who related the security of the QKD to entanglement purification protocols^40^ and Calderbank-Shor-Steane (CSS) codes^41^,^42^ for privacy amplification and error correction.

In our scheme, if the center allows the communication between the two users, she measures her particles with the rectilinear basis. Then, between Bob and Carol, the QKD is equal to a modified EPR scheme^38^,^43^. The users demand that both bit- and phase-flip error rates, *e*_1_ and *e*_2_ of the channel must be sufficiently small,


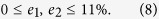


Then, it has been proved that both error rates of the signal are also small enough to allow the CSS code to correct.

We remark that the proof of our scheme is based on the proof of the modified Lo-Chau EPR scheme^38^, and the error correction and privacy amplification procedure that we use are exactly the same as in Shor-Preskill’s proof. The point is the following: Once the error rate is shown to be correctable by a quantum (CSS) code, the procedure for error correction and privacy amplification in their proof can be carried over directly to our scheme.

From the above discussion, we remark that the Ekert QKD scheme is a special case of our scheme where 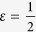. In a general case, however, the bases used by the two users are compatible with a probability (1 − 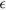)^2^ + 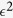, which goes to 1 as 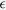 goes to zero, either. From Part B, we know the value of 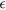 should be small but can not be zero. The main constraint on 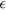 is that there should be enough particles for an accurate estimation of the error rates *e*_1_ and *e*_2_. We assume that *N* pairs of particles are chosen by Alice, i.e., *N* particles are transmitted from Alice to Bob and Carol, respectively. On average, only *N*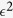 belong to the case where both Bob and Carol choose the diagonal basis. To estimate *e*_2_ reasonably and accurately, one need to make sure that this number *N*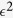 is larger than the some fixed number say *m*_2_. The key point to note is that this number *m*_2_ depends on *e*_2_ and the desires accuracy of the estimation but not on *N*. (Indeed, the number *m*_2_ can be computed from classical statistical analysis). So


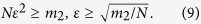


As *N* tends to ∞, 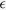 can tend to zero, but never reach it. And the efficiency of this scheme is asymptotic 100%.

### Multiparty QKD Scheme

We now generalize the two-party efficient QKD scheme to a multiparty one. Let us suppose that the center Alice prepares pairs of particles in the certain entangled state:


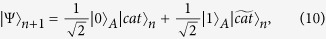


where the *n*-particle entanglement is the cat state 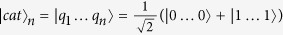, and 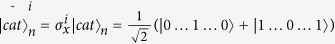 (Here, 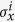 means that a 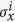 operation is performed on the *i*th qubit of the state |*cat*〉_*n*_ by Alice, and *i* ∈ {1…*n*}), and *n* ≥ 3. She sends a sequence of particles *j* to each party *P*_*j*_ (*j* = 1…*n*) and leaves the corresponding particles A for herself. If she allows the n users to distribute a common key among themselves, Alice measures her particles along the rectilinear basis. Similar to the two-party scheme, each party *P*_*j*_ chooses his bases between the rectilinear and diagonal basis independently with the probability 1 − 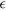 and 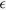 respectively, and performs measurement. After exchanging enough particles, Alice broadcasts over the public channel the sequence of measurement results and on which qubit is put a operator “*σ*_*x*_”. Then, each party also announces the sequence of bases he uses, but not the results that he obtains. There are two cases in which they keep the results of the measurement for a secret key: if all of them choose the rectilinear basis, the bits are kept for establishing a key; if only two of them use the same bases, and the others all use the diagonal basis, the bits are used to ensure the security of the channel connecting with them. Similar to the two-party scheme, to establish a common key, the *i*th user would invert all bits or not based on Alice’s results. Therefore, the users take on an error analysis to detect whether or not there is any eavesdropper. If the error rate is enough smaller, they carry out the last step, which is reconciliation and privacy amplification. Hence, a common key among n parties is established with the total probability (1 − 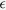)^*n*^. The security of the cryptography network is based on the fundamental postulate of quantum physics that “a nonorthogonal state cannot be cloned”.

Now we choose a three-party scenario by way of example and it will become evident that there are many parties that will work equally well. The scheme works in the following way:

1. The center Alice prepares a sequence of pairs of particles in the four-particle entangled state 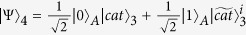, where 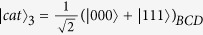, and 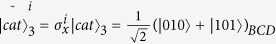 (here, suppose Alice choose that *i* = 2). Then, she sends particles B, C and D to the users Bob, Carol and David, and leaves the corresponding particle A for herself.
2. If she allows to establish a common key among the three parties, she measures her sequence of particles with the rectilinear basis, and then each user chooses his bases between the rectilinear and diagonal basis independently with the probability 1 − 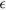 and 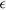, respectively, and performs measurement.
3. After Alice broadcasts her results and the number *i* which equals to 2, and the users also announce their bases, they choose the bits kept for a secret key in two cases. In one case, the users all use the rectilinear basis, they keep the bit as a raw key based on the results of Alice’s measurement. It means that if Alice broadcasts the result “1”, Carol would invert all her bits; else, the users do nothing with the bits. In the other case, one of them chooses the diagonal basis, and the other two use the same bases (rectilinear or diagonal), the bits are used to check the security of the communication channel. From Table 2, we find that, in the case, the bits of the two users which use the same bases are correlated. Therefore, they publicly compare their results of measurement and estimate the error rates. If the error rates are not too large, they proceed to the next steps. Otherwise, they throw away the data and restart the whole procedure. In Table 2, we give an example to explain the refined error analysis. And the key is secret to the center. In one word, the sifted key is generated with the total probability (1 − 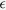)^3^ which goes to 1 as 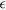 goes to zero. Obviously, the probability 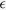 can never be zero (seeing Sec. II).
4. For the first case mentioned above, similar to the two-party scheme, the users randomly pick a fixed number say *m* particles and broadcast their measurement results, then estimate the error rate 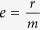, where *r* is the number mismatches. If the constraint *e* < *e*_*max*_, they go to the last step, reconciliation and privacy amplification to reduce the error rate. This error analysis is ensuring the security at the cryptography network against both eavesdropping and intrinsic noise.

## Discussion

In the two-user scheme, pairs of particles for QKD are in the certain entangled state 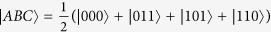. This has density operator 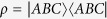.

Tracing out the first qubit, we find the reduced density operator of the system (the second and third qubits),


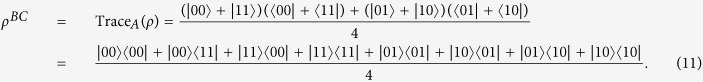


Notice that this state is a mixed state. So if and only if the center Alice measures her particles and announces the measurement results, the two users can distribute a common key. However, once Alice measures her qubits, the left ones are in one of the EPR states (|Φ^+^〉 or |Ψ^+^〉) and then between Bob and Carol, the QKD is equal to a modified EPR scheme^38^,^43^. The key is proved to be unknown to Alice. It is evident that works well in the multiuser scheme. Furthermore, in that multiuser scheme, because of the cyclic symmetry of the state 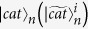, we can check the channel between any two of the users by an error analysis. In summary, we propose a QKD scheme with some certain entanglement of three qubits to solve a special cryptographic task. Combined with the idea presented by Lo *et al*.^17^, the efficiency of this scheme can be increased to tend to 100%. To make the scheme secure against the dominant basis eavesdropping attack, it is crucial to have a refined error analysis in place of a naive error analysis. Then we provide a proof of security of our new QKD scheme against the most general type of attack by generalizing Shor and Preskill’s proof of security of the other schemes^38^. Furthermore, based on the scheme, we have shown that a certain entangled state can be used to distribute a common key among multi-user, in such a way that a quantum cryptography network can be established without any quantum memory. The security of the cryptography network is based on the fundamental postulate of quantum physics that “a nonorthogonal state cannot be cloned”. Also, an error analysis ensure the security of the communication channel between any two of the users.

## Additional Information

**How to cite this article:** Xue, P. *et al*. Efficient multiuser quantum cryptography network based on entanglement. *Sci. Rep.* **7**, 45928; doi: 10.1038/srep45928 (2017).

**Publisher's note:** Springer Nature remains neutral with regard to jurisdictional claims in published maps and institutional affiliations.

## Figures and Tables

**Table 1:** Example of the two-user QKD scheme.

A result	0 0	0 0	0	0 0	0 1 1	1 1	1	1 1	1
B basis	+ +	+ +	×	× ×	× + +	+ +	×	× ×	×
B bit value	0 0	1 1	0	0 1	1 0 0	1 1	0	0 1	1
C basis	+ ×	+ ×	+	× +	× + ×	+ ×	+	× +	×
C bit value	0 0/1	1 0/1	0/1	0 0/1	1 1 0/1	0 0/1	0/1	1 0/1	0
Compatible?	y n	y n	n	y n	y y n	y n	n	y n	y
Sited key	0	1		0	1 0	1		0	1

Here + and × represent the rectilinear 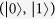 and diagonal bases 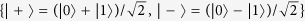, respectively.

**Table 2:** We choose the case by the way of example, in which the measurement results of Alice are “0”, and all of the three users choose the rectilinear basis to establish a common key.

A result	0	0	0	0	0	0	0	0	0	0
B basis	+	+	×	×	×	×	×	×	×	×
B bit value	0	1	0	0	1	1	0	0	1	1
C basis	+	+	+	+	+	+	×	×	×	×
C bit value	0	1	0	1	0	1	0	1	0	1
D basis	+	+	+	+	+	+	×	×	×	×
D bit value	0	1	0	1	0	1	0	1	0	1
Sited key	0	1								

Suppose Bob use the diagonal basis and the other two choose the same ones for an error analysis. If the error rates are not too large (less than *e*_*max*_), the communication channel between Carol and David is proved secure. Since the cyclic symmetry of the GHZ state, it is evident that by the same way we can check the channel between any two of the users.
